# Reliability, Validity, and Comparison of Barbell Velocity Measurement Devices during the Jump Shrug and Hang High Pull

**DOI:** 10.3390/jfmk8010035

**Published:** 2023-03-16

**Authors:** Timothy J. Suchomel, Baylee S. Techmanski, Cameron R. Kissick, Paul Comfort

**Affiliations:** 1 Department of Human Movement Sciences, Carroll University, Waukesha, WI 53186, USA; 2 Directorate of Sport, Exercise, and Physiotherapy, University of Salford, Salford M6 6PU, UK; 3 Athlete Performance, Mequon, WI 53092, USA; 4 New York Mets, Queens, NY 11368, USA

**Keywords:** weightlifting, power clean, velocity-based training, load–velocity profile

## Abstract

This study examined the reliability, potential bias, and practical differences between the GymAware Powertool (GA), Tendo Power Analyzer (TENDO), and Push Band 2.0 (PUSH) during the jump shrug (JS) and hang high pull (HHP) performed across a spectrum of loads. Fifteen resistance-trained men performed JS and HHP repetitions with 20, 40, 60, 80, and 100% of their 1RM hang power clean, and mean (MBV) and peak barbell velocity (PBV) were determined by each velocity measurement device. Least-products regression and Bland–Altman plots were used to examine instances of proportional, fixed, and systematic bias between the TENDO and PUSH compared to the GA. Hedge’s *g* effect sizes were also calculated to determine any meaningful differences between devices. The GA and TENDO displayed excellent reliability and acceptable variability during the JS and HHP while the PUSH showed instances of poor–moderate reliability and unacceptable variability at various loads. While the TENDO and PUSH showed instances of various bias, the TENDO device demonstrated greater validity when compared to the GA. Trivial–small differences were shown between the GA and TENDO during the JS and HHP exercises while trivial–moderate differences existed between GA and PUSH during the JS. However, despite trivial–small effects between the GA and PUSH devices at 20 and 40% 1RM during the HHP, practically meaningful differences existed at 60, 80, and 100%, indicating that the PUSH velocity outputs were not accurate. The TENDO appears to be more reliable and valid than the PUSH when measuring MBV and PBV during the JS and HHP.

## 1. Introduction

Researchers have concluded that weightlifting movements and their derivatives may provide greater strength, power, and speed adaptations compared to other methods of training [1], especially when added to traditional resistance training programs [2]. A growing body of research has shown that training with weightlifting pulling derivatives (i.e., clean or snatch variations that exclude the catch phase) [3] may improve dynamic and isometric strength, jump performance, sprint speed, and change of direction performance [4,5,6,7]. This is due to the performance of these movements providing effective force and velocity overload stimuli via triple extension of the hip, knee, and ankle (plantarflexion) joints [3,8,9]. Specifically, some pulling derivatives allow for the use of loads greater than an individual’s one-repetition maximum (1RM) during a catching variation [10,11,12,13]. In contrast, other pulling derivatives allow practitioners to effectively prescribe lighter loads with more ballistic exercises (e.g., jump shrug [JS] and hang high pull [HHP]) to emphasize velocity characteristics without sacrificing intent [14,15,16,17,18,19,20].

While some ballistic exercises may be prescribed using percentages of the 1RM of biomechanically similar movement (e.g., jump squat loaded based on back squat) [21,22], a common issue identified with prescribing weightlifting pulling derivatives is based around how to load the exercises. For example, researchers typically investigate pulling derivatives using percentages of an individual’s 1RM performed during a catching variation of either the clean or snatch [5,6,7,10,11,14,15,16,17,18,19,20,23,24,25,26,27,28,29,30,31]. While this may not be an issue for practitioners who prescribe both catching and pulling derivatives, those who prescribe only the latter may find it difficult to define submaximal, near maximal, and maximal loads. Some researchers have investigated and discussed using percentages of an individual’s body mass to prescribe pulling derivatives [32,33]. While prescribing loads in this manner may seem appealing, this method fails to account for differences in relative strength, which may lead to either over- or under-prescribing loads for a group of individuals. Thus, other alternatives for load prescription methods should be investigated.

A method of training that has gained popularity within the last decade has been termed velocity-based training. This method of load prescription uses technology to measure the velocity of a movement (typically barbell velocity) during each exercise repetition to determine whether or not an individual is meeting the desired training goal within a session or phase of training [34,35]. While the vast majority of the velocity-based training literature has focused on comparisons with other loading methods [36,37], load prescription [38,39], or the reliability and validity of various velocity devices [40,41,42,43], researchers have primarily focused on traditional resistance training exercises such as the back squat and bench press. In contrast, limited research has investigated the use of velocity measurement devices with weightlifting derivatives [44,45]. Due to the potential of using velocity to prescribe loads for weightlifting pulling derivatives, either via prediction of 1RM performance or the use of velocity thresholds, further research is needed to determine the reliability and validity of such devices. Therefore, the purposes of this study were to examine the reliability, potential bias, and practical differences between three velocity measurement devices during two velocity-dominant weightlifting pulling derivatives performed across a spectrum of loads. It was hypothesized that all the devices would be reliable but show some instances of either proportional, fixed, or systematic bias. Based on pilot testing, it was further hypothesized that small–moderate differences in the mean (MBV) and peak barbell velocity (PBV) would exist when compared to the criterion measurement.

## 2. Materials and Methods

### 2.1. Participants

Fifteen resistance-trained men with previous hang power clean (HPC) experience participated in this study (age = 25.5 ± 4.5 years, body mass = 88.3 ± 15.4 kg, height = 176.1 ± 8.5 cm, relative one repetition maximum (1RM) HPC = 1.3 ± 0.2 kg/kg, and relative 1RM back squat = 2.03 ± 0.3 kg/kg). Prior to the study, the participants had been consistently training at least three times per week for the last year. This study was conducted according to the guidelines of the Declaration of Helsinki and was approved by the Carroll University institutional review board (#19-015; approved 15 April 2019). Each participant read and signed an informed consent form prior to their participation.

### 2.2. Design 

A repeated measures, cross-sectional design was used to examine the reliability, relationships, and differences between three different barbell velocity measurement devices during the JS and HHP performed across a spectrum of loads. Each participant attended three testing sessions over two weeks that included a 1RM HPC, a JS testing session, and HHP testing session. The JS and HHP were performed using percentages of the participant’s 1RM HPC.

### 2.3. 1RM Hang Power Clean and Exercise Familiarization

Each participant performed a 1RM HPC during their first testing session using previously discussed procedures [16,28,29]. Briefly, upon arrival for the 1RM HPC session, the participants were weighed and then completed general (light–moderate cycling and dynamic stretching) and specific (sets of the HPC using 30, 50, 70, and 90% of their predicted 1RM) exercises. Following the warm-up sets, the participants performed maximum attempts until a 1RM was reached. A minimum 2.5 kg increase was required between attempts and the participant was required to perform the lift without the top of their thighs dropping below parallel during the catch phase. This was visually monitored by principal investigator and other testers. No more than four attempts were required to achieve a 1RM. After the 1RM was achieved and a self-selected rest period was provided, the participants were familiarized with the JS and HHP exercises by performing lightly loaded repetitions (<50% 1RM HPC). The coaching cues provided were based on those previously discussed within the literature [46,47].

### 2.4. Exercise Testing Sessions

Each participant returned to the laboratory on two separate occasions to perform sets of either the JS or HHP exercise. The first session took place one week following the 1RM HPC session and the subsequent session followed 48 h later. The order of the JS and HHP testing sessions was randomized for each participant in an effort to prevent an order effect. Upon arriving for the testing session, each participant performed the same general and dynamic warm-up described above before performing a self-selected warm-up with an empty 20 kg barbell. The participant then performed an exercise-specific warm-up that included a set of three repetitions of the testing exercise (i.e., JS or HHP) with 30 and 50% of their 1RM HPC [16,28,29,30]. Following the warm-up, participants were given a two-minute rest period while their first testing load was placed on the barbell. Participants then performed three repetitions each of either the JS or HHP with 20, 40, 60, 80, and 100% of their 1RM HPC in a progressive order. A progressive load order was chosen to increase the ecological validity of the collected data. Each repetition was performed based on previous descriptions [18,20,46,47], whereby the participant stood in the mid-thigh (power) position, received a countdown of “3, 2, 1, Go!”, performed a hip hinge movement where they lowered the barbell to a position just above their patellae and, without pausing, transitioned back to the mid-thigh position flexing their knees and elevating the barbell back up their thighs, and rapidly extended their hip, knee, and ankle (plantarflexion) joints to perform the 2nd pull phase of weightlifting derivatives. The primary difference was that the participant either jumped as high as possible or elevated the barbell to chest height by flexing the elbows during the JS [46] and HHP [47], respectively. One minute of rest was provided between each repetition while two minutes of rest were provided between loads. The participants were allowed to use lifting straps during the repetitions at 100% 1RM HPC to minimize the impact of grip strength with a maximal load. 

### 2.5. Data Analyses

**Figure 1:**
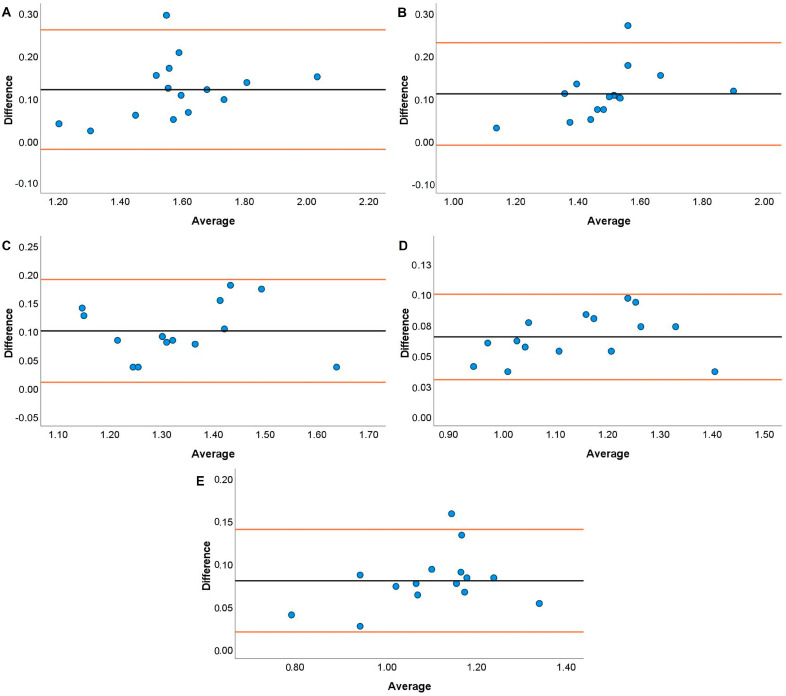
Bland–Altman plots showing variation of the Tendo Power Analyzer compared to the GymAware Powertool for mean barbell velocity during the jump shrug performed with loads of 20 (**A**), 40 (**B**), 60 (**C**), 80 (**D**), and 100% (**E**) of participants’ 1RM hang power clean. The black line displays the mean systematic bias, and the orange lines represent the 95% confidence intervals.

Mean (MBV) and peak barbell velocity (PBV) during each JS and HHP repetition were measured concurrently using a GymAware Powertool ((GA) Kinetic Performance Technology, Braddon, Australia), Tendo Power Analyzer ((TENDO) Tendo Sports Machines, London, UK), and Push Band 2.0 ((PUSH) Push Inc., Toronto, ON, Canada). To prevent disruption to the participant’s grip and displacement measurement, the Push Band 2.0 (PUSH) was placed on the inside of the collar while the GA and TENDO tethers were attached on the sleeve of the barbell (Figure 1). The GA and PUSH devices were connected via Bluetooth to tablet (iPad 2, Apple Inc., Cupertino, CA, USA) and mobile phone (iPhone 7S, Apple Inc., Cupertino, CA, USA) with the latest GA and PUSH application versions, respectively. In contrast, the TENDO was attached through a wired connection where the velocity information was displayed on the device’s computer. The GA used a variable rate sampling with level crossing detection while the TENDO used a factory setting minimum detectable threshold filter of 35 cm for sampling. Finally, the PUSH device was sampled at 200 Hz based on factory settings. Briefly, the GA and TENDO determined the JS and HHP barbell velocities by measuring the vertical displacement determined by the rotational movement of their respective cable and spool designs. MBV and PBV were then determined by dividing the calculated displacement by the movement time. The primary difference between the devices is that the GA also possess a sensor to measure the angle of the cable to adjust for horizontal displacement of the barbell. The PUSH device uses a 3-axis accelerometer and gyroscope within the device that provide six degrees of freedom. The PUSH device was set to “Bar Mode” and used the factory algorithms based on the selected exercises to determine the MBV and PBV during the concentric phase of each movement.

### 2.6. Statistical Analyses

The Shapiro–Wilks test was used to examine the normality of data distribution of each variable. Two-way mixed intraclass correlation coefficients and typical error expressed as coefficients of variation were used to examine the relative and absolute reliability of each device, respectively. The intraclass correlation coefficients were interpreted as poor, moderate, good, and excellent if magnitudes were <0.50, 0.50–0.74, 0.75–0.90, and >0.90, respectively [48]. In addition, acceptable coefficients of variation were classified as <10% [49]. Because previous researchers demonstrated that the GA showed the greatest validity compared to 3D motion analysis during the power clean [45], the GA was used as the criterion measurement in the current study. Coefficients of determination (R^2^) were used to examine the association of the TENDO and PUSH devices against the GA. In addition, fixed and proportional bias compared to the GA device was determined using previously described methods with ordinary least-products regression [45,50,51]. Using this method, fixed bias was present if the 95% confidence interval for the intercept (x) did not include 0. In addition, proportional bias was present if the 95% confidence interval for the slope (y) did not include 1.0. Systematic bias between the GA and the TENDO and PUSH devices was then determined using 95% limits of agreement. Finally, Hedge’s g effect sizes were used to provide a measure of practical significance between the devices. Effect sizes magnitudes of 0.00–0.19, 0.20–0.59, 0.60–1.19, 1.20–1.99, 2.00–3.99, and ≥4.00 were interpreted as trivial, small, moderate, large, very large, and nearly perfect, respectively [52]. All statistical tests were completed using SPSS 28 (IBM, Chicago, IL, USA).

## 3. Results

All MBV and PBV data were normally distributed. The GA and TENDO devices displayed excellent reliability and acceptable variability for all MBV and PBV data (Table 1). In contrast, while the PUSH device generally showed good–excellent reliability and acceptable variability during the JS, moderate reliability and larger, unacceptable variability (13.2%) was present during the 100% load for PBV and MBV, respectively. In addition, the PUSH device also showed poor–moderate reliability of PBV during the 20 and 40% 1RM loads while larger and unacceptable variability (11.2% and 12.0%) was shown with MBV and PBV at 40% 1RM.

### 3.1. Jump Shrug

The least-products regression statistics for the JS are displayed in Table 2. Apart from small proportional bias of MBV at 20% 1RM, the TENDO did not display any proportional or fixed bias during the JS. In addition, R^2^ values ranged from 0.88 to 0.98 and 0.87 to 0.98 for MBV and PBV, respectively. While the PUSH device did not show any proportional or fixed bias during the JS, R^2^ values were considerably lower compared to the TENDO with values ranging from 0.47 to 0.86 and 0.58 to 0.89 for MBV and PBV, respectively.

**Figure 2:**
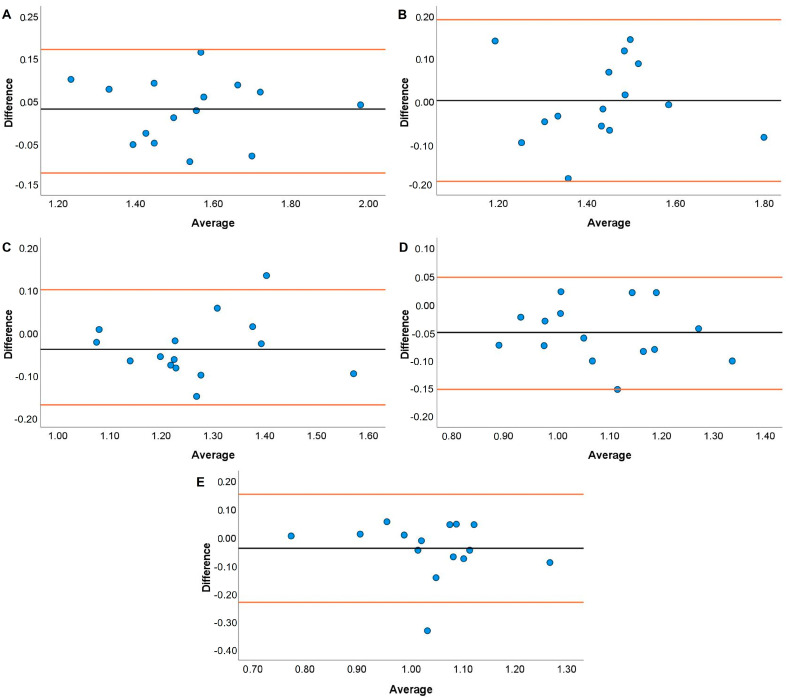
Bland–Altman plots showing variation of the Push Band 2.0 compared to the GymAware Powertool for mean barbell velocity during the jump shrug performed with loads of 20 (**A**), 40 (**B**), 60 (**C**), 80 (**D**), and 100% (**E**) of participants’ 1RM hang power clean. The black line displays the mean systematic bias, and the orange lines represent the 95% confidence intervals.

**Figure 3:**
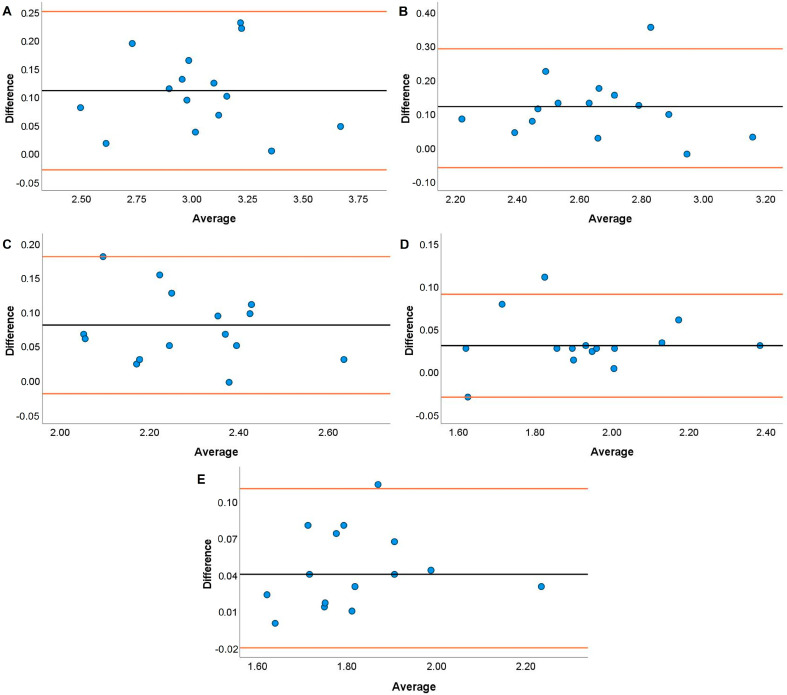
Bland–Altman plots showing variation of the Tendo Power Analyzer compared to the GymAware Powertool for peak barbell velocity during the jump shrug performed with loads of 20 (**A**), 40 (**B**), 60 (**C**), 80 (**D**), and 100% (**E**) of participants’ 1RM hang power clean. The black line displays the mean systematic bias, and the orange lines represent the 95% confidence intervals.

**Figure 4:**
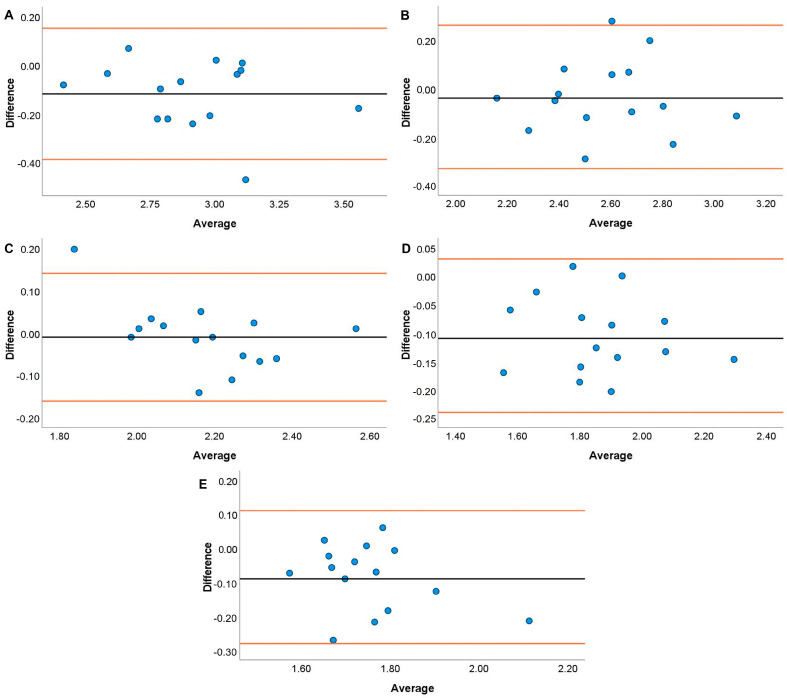
Bland–Altman plots showing variation of the Push Band 2.0 compared to the GymAware Powertool for peak barbell velocity during the jump shrug performed with loads of 20 (**A**), 40 (**B**), 60 (**C**), 80 (**D**), and 100% (**E**) of participants’ 1RM hang power clean. The black line displays the mean systematic bias, and the orange lines represent the 95% confidence intervals.

JS systematic bias comparisons for the TENDO and PUSH with the GA are displayed in Figure 1, Figure 2, Figure 3 and Figure 4. The TENDO showed some systematic bias by overestimating MBV during the JS with loads of 20, 40, and 100% 1RM. Similarly, PBV was also overestimated at loads of 40, 80, and 100% 1RM. The PUSH device also showed systematic bias by overestimating MBV at 60 and 80% 1RM but underestimating MBV at 100% 1RM. In addition, the PUSH device also overestimated PBV at 40 and 60% 1RM and underestimated PBV at 20% 1RM. 

### 3.2. Hang High Pull

The least-products regression statistics for the HHP are displayed in Table 3. The TENDO device displayed proportional and fixed bias with MBV at 100 and PBV at 20% 1RM, respectively, during the HHP exercise. However, R^2^ values ranged from 0.93 to 0.96 and 0.79 to 0.94 for MBV and PBV, respectively. Proportional and fixed bias was displayed by the PUSH device for MBV at 20, 40, and 60% 1RM during the HHP. In addition, fixed bias was shown for PBV during 100% 1RM. The R^2^ values for the PUSH device ranged from 0.77 to 0.96 and 0.62 to 0.92 for MBV and PBV, respectively.

**Figure 5:**
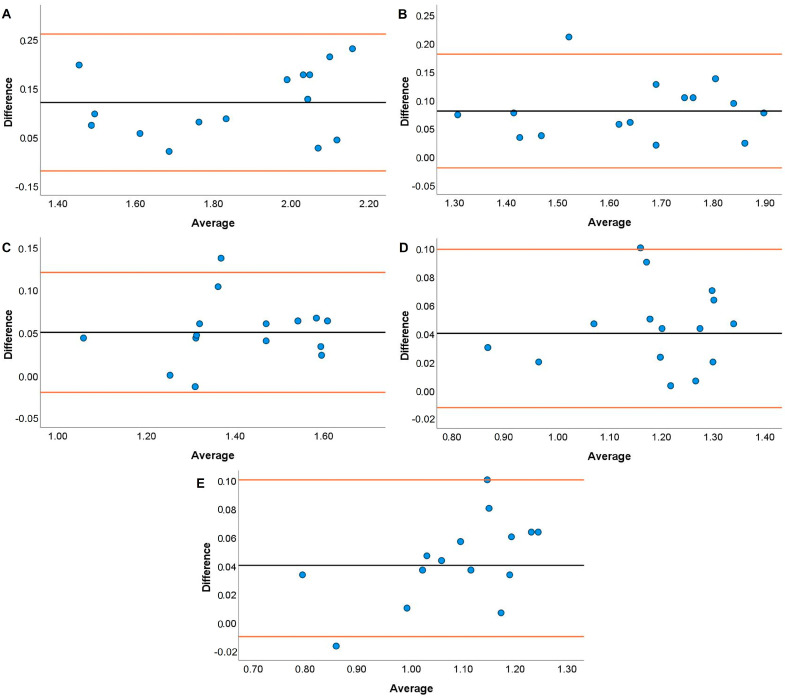
Bland–Altman plots showing variation of the Tendo Power Analyzer compared to the GymAware Powertool for mean barbell velocity during the hang high pull performed with loads of 20 (**A**), 40 (**B**), 60 (**C**), 80 (**D**), and 100% (**E**) of participants’ 1RM hang power clean. The black line displays the mean systematic bias, and the orange lines represent the 95% confidence intervals.

**Figure 6:**
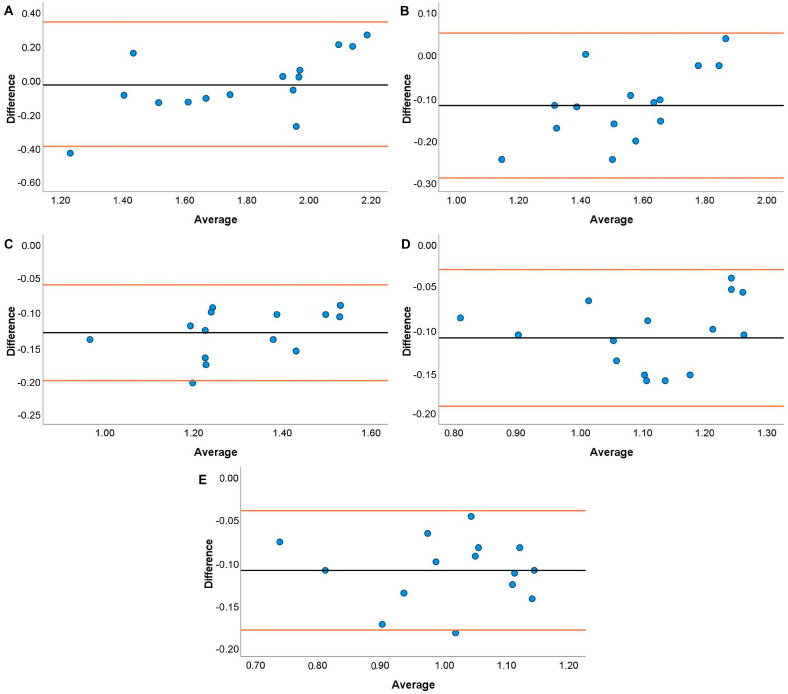
Bland–Altman plots showing variation of the Push Band 2.0 compared to the GymAware Powertool for mean barbell velocity during the hang high pull performed with loads of 20 (**A**), 40 (**B**), 60 (**C**), 80 (**D**), and 100% (**E**) of participants’ 1RM hang power clean. The black line displays the mean systematic bias, and the orange lines represent the 95% confidence intervals.

**Figure 7:**
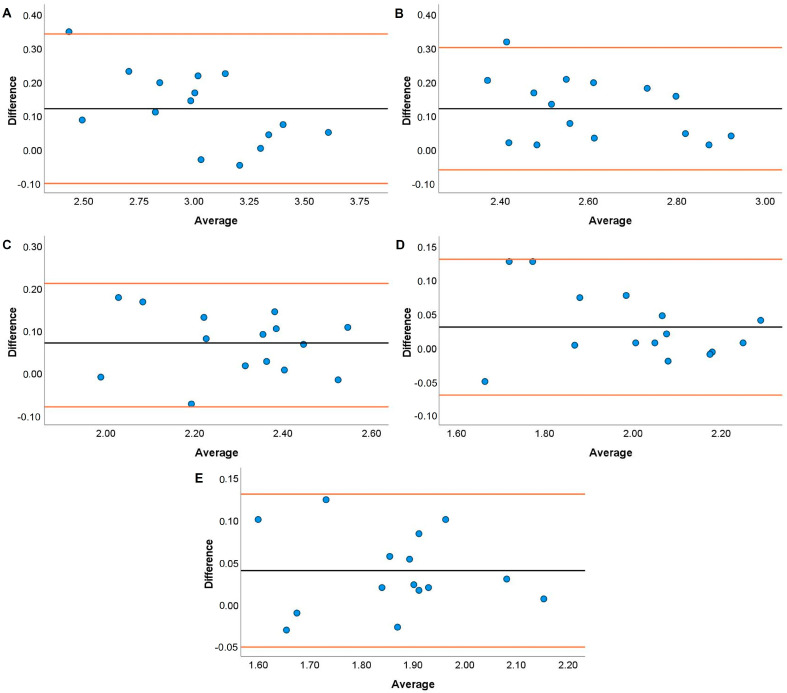
Bland–Altman plots showing variation of the Tendo Power Analyzer compared to the GymAware Powertool for peak barbell velocity during the hang high pull performed with loads of 20 (**A**), 40 (**B**), 60 (**C**), 80 (**D**), and 100% (**E**) of participants’ 1RM hang power clean. The black line displays the mean systematic bias, and the orange lines represent the 95% confidence intervals.

**Figure 8:**
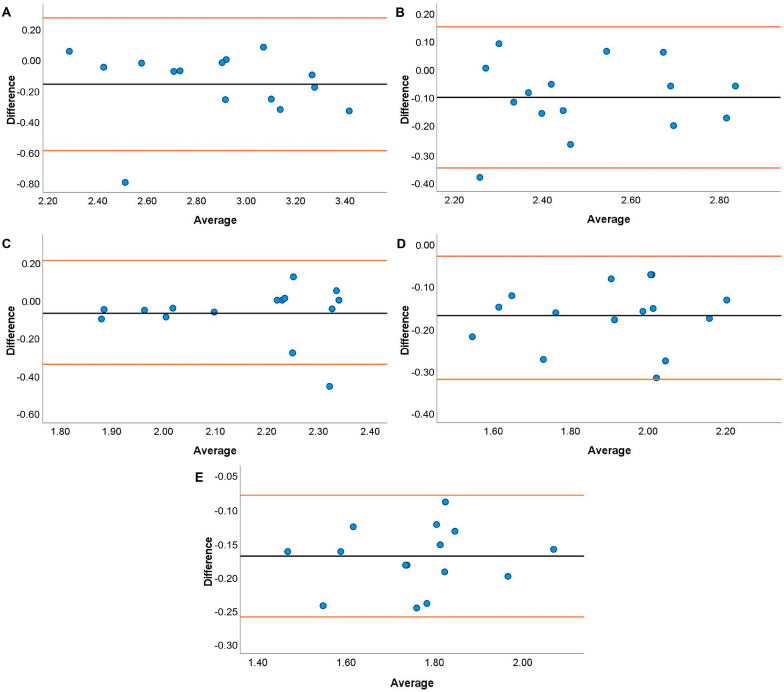
Bland–Altman plots showing variation of the Push Band 2.0 compared to the GymAware Powertool for peak barbell velocity during the hang high pull performed with loads of 20 (**A**), 40 (**B**), 60 (**C**), 80 (**D**), and 100% (**E**) of participants’ 1RM hang power clean. The black line displays the mean systematic bias, and the orange lines represent the 95% confidence intervals.

Systematic bias analyses for the HHP are displayed in Figure 5, Figure 6, Figure 7 and Figure 8. Systematic bias was present for the TENDO as it overestimated MBV and PBV at 20, 40, and 100% 1RM and 40, 80, and 100% 1RM, respectively. In contrast, the PUSH displayed systematic bias by underestimating MBV and PBV. Specifically, systematic bias for MBV was shown at loads of 20, 60, and 100% 1RM, while bias for PBV was shown at loads of 20, 40, and 60% 1RM.

### 3.3. Effect Size Comparisons

The effect size magnitudes between the TENDO and PUSH devices and the GA are displayed in Table 4 and Table 5. Small–moderate and trivial–small effects existed between the TENDO and GA for MBV and PBV during the JS, respectively. In addition, small and trivial–moderate effects existed between the TENDO and GA for MBV and PBV during the HHP, respectively. None of the differences between the TENDO and GA were practically meaningful as the confidence intervals included zero.

Trivial–small and trivial–moderate effects existed between the PUSH and GA for MBV and PBV during the JS, respectively. In addition, trivial–moderate and small–moderate effects existed between the PUSH and GA for MBV and PBV during the HHP, respectively. It should be noted, however, that the MBV and PBV measured by the PUSH compared to the GA at 60, 80, and 100% 1RM were practically meaningful as indicated by confidence intervals that did not include zero. None of the other differences between the PUSH and GA were practically meaningful.

## 4. Discussion

The current study examined the reliability, validity, and bias of the TENDO and PUSH devices compared to the GA during the JS and HHP performed with a spectrum of loads. The primary findings of the current study are as follows. First, excellent test–retest reliability with acceptable variability for MBV and PBV was present for the GA and TENDO during both the JS and HHP exercises while the PUSH reliability ranged from poor to excellent with acceptable or larger variability depending on the exercise and/or load being examined. Second, both the TENDO and PUSH devices showed some instances of proportional, fixed, and systematic bias; however, the validity of the TENDO appeared to be greater than the PUSH device. Finally, while there were no practically meaningful differences between the TENDO and GA for MBV and PBV measured during the JS or HHP, practically meaningful differences existed between the PUSH and GA during the HHP exercise, but not for the JS.

Previous researchers indicated that the TENDO provided reliable and valid measurements of average concentric velocity during the back squat exercise but displayed significantly different peak concentric velocities compared to the criterion 3D motion capture system [53]. Excellent reliability and acceptable variability for MBV and PBV during the JS and HHP performed across all loads were shown by the TENDO in the current study. While the PUSH device showed similar trends in reliability and variability as the TENDO during the JS, moderate test–retest reliability and a larger magnitude of variability was shown at 100% 1RM for PBV and MBV. Further instances of poor–moderate reliability and unacceptable variability (>10%) [49] were shown by the PUSH device for PBV at 20 and 40% 1RM and MBV and PBV at 40% 1RM. It should be noted that Lake et al. [54] suggested that MBV may be questioned when using the PUSH device after examining the bench press exercise. Although not directly applicable to the current study, it appears that the PUSH device may not provide consistently reliable MBV or PBV during the JS and HHP exercises either. While this is the first study to examine the reliability of the TENDO and PUSH devices during the JS and HHP, both exercises may be classified as velocity-dominant weightlifting pulling derivatives [3,8,9]. Thus, further research may be warranted with pulling derivatives that possess smaller displacements (i.e., force-dominant pulling derivatives such as a hang pull or countermovement shrug) to determine if similar trends in reliability are present for the TENDO and PUSH devices.

Both the TENDO and PUSH devices showed some instances of either proportional, fixed, or systematic bias when compared to the GA during both the JS and HHP. The only instance of proportional bias during the JS was shown by the TENDO when measuring MBV at 20% 1RM. Despite this occurrence, the TENDO R^2^ values for MBV and PBV tended to be much higher across loads compared to the PUSH device, especially at the heaviest load examined (i.e., 100% 1RM). It should be noted that the TENDO showed small systematic bias by overestimating MBV at 20, 40, and 100% 1RM and PBV at 40, 80, and 100% 1RM during the JS. However, the PUSH device showed a lack of consistency where systematic bias was shown by both overestimating MBV at 60 and 80% 1RM and PBV at 40 and 60% 1RM and underestimated MBV at 100% 1RM and PBV at 20% 1RM. It is not entirely surprising that both the TENDO and PUSH devices demonstrated some instances of systematic bias due to the ability of the GA to account for horizontal displacement during resistance training exercises [55]. While the JS is a ballistic weightlifting pulling derivative, the mechanics of movement slightly differ from vertical jump variations that are performed almost entirely in the vertical plane [46,56]. Thus, differences are to be expected with a linear position transducer that does not account for any horizontal displacement (i.e., TENDO) or an inertial measurement unit whose algorithm may very between ballistic exercises (i.e., PUSH). Similar to the current findings, Lake and colleagues [50] indicated that the PUSH device did not demonstrate any fixed or proportional bias during the countermovement jump. However, the previous authors noted that peak and mean velocity were overestimated compared to a 3D motion analysis system. It should be noted that the previous study used the PUSH device in “Jump Mode”, while the present study used “Bar Mode” when examining velocity characteristics. Given the unique nature of the exercise, researchers could consider comparing the JS performed using both modes. 

The TENDO and PUSH devices showed several instances of bias during the HHP exercise. Specifically, proportional and fixed bias were present with the TENDO for MBV at 100% 1RM and PBV at 20%. In contrast, proportional and fixed bias for MBV were present with the PUSH device at 20, 40, and 60% 1RM while fixed bias was also shown for PBV at 100% 1RM. Like the JS, greater variance for both MBV and PBV was accounted for with the TENDO versus the PUSH as demonstrated by the R^2^ values. Interestingly, the TENDO tended to overestimate MBV and PBV while the PUSH underestimated both variables compared to the GA during the HHP. Again, these instances of systematic bias may be due to each device’s characteristics in how they calculate MBV and PBV. The HHP is a unique weightlifting derivative that requires an individual to elevate the barbell to their chest height [47], often surpassing the required displacement to perform an HPC catch. As a result, it is important that velocity measurement devices can account for large displacements when calculating MBV and PBV. As shown by the effect size comparisons above, the PUSH device may not have the capacity to do this, especially at heavier loads.

A unique aspect of the current study was the calculation of effect size magnitudes when comparing the MBV and PBV produced by the GA and the TENDO and PUSH. In general, small–moderate and trivial–small differences were shown for MBV and PBV when comparing the GA and TENDO during both the JS and HHP exercises. While trivial–small and trivial–moderate effects existed when comparing MBV and PBV, respectively, between the GA and PUSH during the JS, these trends were not followed during the HHP. Trivial–small effects for MBV and PBV existed when comparing the GA and PUSH devices at 20 and 40% 1RM during the HHP; however, these magnitudes became much larger at loads of 60% 1RM and higher, indicating that the velocity outputs of the PUSH device were meaningfully different and not accurate. This is an important finding given the use of different velocity measurement devices within strength and conditioning settings. 

Using the GA device as a criterion measurement for comparison may be viewed as a potential limitation of the current study. It should be noted, however, that authors of a recent systematic review concluded that the GA shows the greatest accuracy of available devices when it has been directly compared to the gold standard of 3D high-speed motion capture [55]. Another potential limitation is the lack of literature to compare the current findings to. To the authors’ knowledge, this is the first study to examine the reliability and validity of different velocity measurement devices during the JS and HHP exercises. Therefore, it is recommended that researchers and practitioners interested in using velocity-based training and monitoring with weightlifting movements examine the current derivatives as well as others typically used in training. Finally, it is important to note that the findings of the current study may not be generalized to other populations. Thus, researchers and practitioners should interpret the current findings with caution until further research is conducted.

## 5. Conclusions

The results of the current study indicate that the TENDO appears to be more reliable and valid than the PUSH when measuring MBV and PBV during the JS and HHP. Although the TENDO tends to overestimate velocity metrics, this can be accounted for if the device is consistently used. It should also be noted that velocity measurement devices should not be used interchangeably when measuring MBV and PBV during these exercises. However, strength and conditioning practitioners should also be aware of its limitation to account for horizontal displacement. Thus, it is recommended that the necessary time is dedicated to ensuring proficient exercise technique. Our results also indicate that the PUSH device may underestimate MBV and PBV compared to the GA during the HHP, and in some instances provide inaccurate velocity metrics at higher loads. Although researchers have suggested implementing the HHP with lighter loads (e.g., <50% 1RM) [19,20,28,29], practitioners must consider the limitations of the PUSH and other devices when measuring velocity. From a practical standpoint, it appears that both the GA and TENDO may be used to reliably assess MBV and PBV during both the JS and HHP across an entire loading spectrum. However, further research is needed to evaluate the reliability and validity of these devices using other weightlifting derivatives.

## Figures and Tables

**Table 1:** Test–retest reliability for the GymAware (GA), Tendo unit (TENDO), and Push Band 2.0 (PUSH) devices during the jump shrug and hang high pull.

Load (%)	Jump Shrug				Hang High Pull			
	ICC		CV%		ICC		CV%	
	MBV	PBV	MBV	PBV	MBV	PBV	MBV	PBV
GA								
20	0.91 (0.78–0.97)	0.97 (0.93–0.99)	7.1 (5.2–11.5)	3.4 (2.5–5.4)	0.95 (0.89–0.98)	0.96 (0.92–0.99)	5.8 (4.2–9.2)	4.6 (3.4–7.4)
40	0.96 (0.91–0.99)	0.97 (0.93–0.99)	4.1 (3.0–6.5)	3.0 (2.2–4.7)	0.97 (0.93–0.99)	0.95 (0.87–0.98)	3.1 (2.3–5.0)	3.1 (2.3–5.0)
60	0.96 (0.89–0.98)	0.97 (0.92–0.99)	3.6 (2.6–5.7)	2.1 (1.5–3.3)	0.96 (0.90–0.98)	0.95 (0.89–0.98)	4.6 (3.3–7.3)	3.0 (2.2–4.8)
80	0.95 (0.87–0.98)	0.96 (0.90–0.99)	4.9 (3.6–7.8)	3.4 (2.5–5.5)	0.98 (0.94–0.99)	0.98 (0.96–0.99)	3.2 (2.3–5.1)	2.5 (1.8–3.9)
100	0.97 (0.93–0.99)	0.96 (0.89–0.98)	3.8 (2.8–6.0)	3.3 (2.4–5.3)	0.98 (0.95–0.99)	0.98 (0.95–0.99)	3.2 (2.3–5.1)	2.2 (1.6–3.5)
TENDO								
20	0.94 (0.85–0.98)	0.98 (0.94–0.99)	5.9 (4.3–9.4)	2.5 (1.8–4.0)	0.97 (0.92–0.99)	0.92 (0.82–0.97)	5.5 (4.0–8.8)	6.0 (4.4–9.7)
40	0.95 (0.87–0.98)	0.97 (0.94–0.99)	5.0 (3.7–8.1)	2.5 (1.8–4.0)	0.97 (0.93–0.99)	0.93 (0.83–0.97)	3.1 (2.3–5.0)	2.9 (2.1–4.6)
60	0.96 (0.90–0.98)	0.93 (0.84–0.98)	3.4 (2.5–5.5)	2.9 (2.1–4.6)	0.96 (0.90–0.99)	0.94 (0.86–0.98)	3.9 (2.9–6.3)	3.3 (2.4–5.3)
80	0.97 (0.93–0.99)	0.96 (0.90–0.98)	3.8 (2.8–6.1)	3.6 (2.6–5.7)	0.96 (0.92–0.99)	0.97 (0.93–0.99)	4.0 (2.9–6.4)	2.9 (2.1–4.7)
100	0.98 (0.97–0.99)	0.95 (0.88–0.98)	2.7 (2.0–4.4)	3.6 (2.7–5.8)	0.97 (0.92–0.99)	0.96 (0.90–0.99)	4.2 (3.0–6.6)	3.1 (2.3–5.0)
PUSH								
20	0.91 (0.77–0.97)	0.96 (0.91–0.99)	5.8 (4.2–9.3)	3.0 (2.2–4.8)	0.90 (0.75–0.96)	0.65 (0.14–0.88)	5.0 (3.6–8.0)	5.1 (3.7–8.2)
40	0.90 (0.77–0.96)	0.96 (0.91–0.99)	6.1 (4.4–9.8)	3.4 (2.5–5.4)	0.88 (0.73–0.96)	0.38 (−0.47–0.78)	11.2 (8.1–18.3)	12.0 (8.6–19.7)
60	0.89 (0.73–0.96)	0.89 (0.75–0.96)	5.5 (4.0–8.9)	4.0 (2.9–6.4)	0.94 (0.85–0.98)	0.90 (0.76–0.96)	6.3 (4.6–10.1)	5.5 (4.0–8.8)
80	0.94 (0.86–0.98)	0.96 (0.91–0.99)	5.3 (3.8–8.5)	3.4 (2.4–5.3)	0.94 (0.84–0.98)	0.88 (0.70–0.96)	6.2 (4.5–9.9)	6.3 (4.6–10.3)
100	0.79 (0.49–0.92)	0.69 (0.26–0.89)	13.2 (9.5–21.5)	8.6 (6.3–14.0)	0.94 (0.86–0.98)	0.92 (0.81–0.97)	5.3 (3.9–8.5)	4.7 (3.4–7.5)

Notes: Load = % 1RM hang power clean; ICC = two-way mixed intraclass correlation coefficient; CV% = typical error expressed as a coefficient of variation percentage; MBV = mean barbell velocity; PBV = peak barbell velocity.

**Table 2:** Least-products regression for the Tendo Power Analyzer (TENDO) and Push Band 2.0 (PUSH) during the jump shrug compared to GymAware Powertool.

Load (%)	R^2^		Slope (95% CI)		Intercept (95% CI)	
	MBV	PBV	MBV	PBV	MBV	PBV
TENDO						
20	0.89	0.94	0.882 (0.767–0.998) †	1.007 (0.866–1.148)	0.074 (−0.124–0.272)	−0.128 (−0.550–0.293)
40	0.90	0.87	0.850 (0.674–1.027)	1.024 (0.804–1.244)	0.123 (−0.153–0.398)	−0.181 (−0.759–0.396)
60	0.88	0.91	0.977 (0.724–1.231)	1.073 (0.929–1.218)	−0.068 (−0.413–0.277)	−0.246 (−0.592–0.100)
80	0.98	0.98	0.959 (0.877–1.040)	0.988 (0.887–1.090)	−0.016 (−0.108–0.076)	−0.010 (−0.220–0.201)
100	0.95	0.96	0.920 (0.795–1.045)	0.970 (0.855–1.086)	0.011 (−0.123–0.145)	0.011 (−0.196–0.217)
PUSH						
20	0.84	0.78	1.009 (0.837–1.181)	1.117 (0.693–1.541)	−0.042 (−0.309–0.226)	−0.213 (−1.334–0.908)
40	0.64	0.66	1.006 (0.610–1.402)	0.994 (0.691–1.297)	−0.005 (−0.575–0.565)	0.051 (−0.690–0.791)
60	0.75	0.89	0.949 (0.697–1.202)	1.042 (0.845–1.238)	0.100 (−0.194–0.394)	−0.018 (−0.432–0.395)
80	0.86	0.89	1.064 (0.901–1.227)	1.054 (0.900–1.207)	−0.016 (−0.184–0.152)	0.008 (−0.283–0.300)
100	0.47	0.58	1.152 (0.926–1.378)	1.248 (0.992–1.504)	−0.114 (−0.378–0.150)	−0.340 (−0.790–0.109)

Notes: Load = % 1RM hang power clean; MBV = mean barbell velocity; PBV = peak barbell velocity; † = proportional bias present.

**Table 3:** Least-products regression for the Tendo Power Analyzer (TENDO) and Push Band 2.0 (PUSH) during the hang high pull compared to the GymAware Powertool.

Load (%)	R^2^		Slope (95% CI)		Intercept (95% CI)	
	MBV	PBV	MBV	PBV	MBV	PBV
TENDO						
20	0.93	0.93	0.921 (0.755–1.087)	1.215 (0.982–1.449)	0.033 (−0.283–0.350)	−0.785 (−1.522–−0.048) ‡
40	0.93	0.79	0.982 (0.888–1.076)	1.214 (0.959–1.470)	−0.052 (−0.208–0.104)	−0.693 (−1.398–0.012)
60	0.95	0.83	0.973 (0.819–1.127)	1.064 (0.797–1.331)	−0.013 (−0.253–0.227)	−0.217 (−0.849–0.415)
80	0.96	0.94	0.978 (0.890–1.066)	1.094 (0.898–1.290)	−0.017 (−0.122–0.088)	−0.220 (−0.630–0.189)
100	0.96	0.91	0.886 (0.791–0.981) †	1.029 (0.842–1.216)	0.082 (−0.022–0.187)	−0.092 (−0.457–0.273)
PUSH						
20	0.77	0.65	0.681 (0.442–0.921) †	1.059 (0.617–1.501)	0.592 (0.149–1.034) ‡	−0.008 (−1.321–1.305)
40	0.89	0.65	0.779 (0.604–0.954) †	0.948 (0.661–1.235)	0.446 (0.185–0.708) ‡	0.226 (−0.501–0.953)
60	0.96	0.62	0.905 (0.825–0.985) †	1.009 (0.689–1.328)	0.247 (0.141–0.353) ‡	0.121 (−0.534–0.777)
80	0.91	0.87	0.953 (0.752–1.155)	0.971 (0.838–1.104)	0.155 (−0.062–0.372)	0.223 (−0.011–0.458)
100	0.91	0.92	1.010 (0.798–1.222)	0.966 (0.832–1.100)	0.101 (−0.111–0.312)	0.230 (0.012–0.448) ‡

Notes: Load = % 1RM hang power clean; MBV = mean barbell velocity; PBV = peak barbell velocity; † = proportional bias present; ‡ = fixed bias present.

**Table 4:** Effect size magnitudes (*g*) and 95% confidence intervals for the Tendo Power Analyzer (TENDO) and Push Band 2.0 (PUSH) during the jump shrug compared to the GymAware Powertool (GA).

**Load (%)**	**MBV**				
	**TENDO**	**PUSH**	**GA**	**TENDO-GA *g***	**PUSH-GA *g***
20	1.64 ± 0.21	1.55 ± 0.18	1.53 ± 0.19	0.59 (−0.14–1.32)	0.15 (−0.57–0.86)
40	1.55 ± 0.18	1.44 ± 0.15	1.44 ± 0.15	0.64 (−0.10–1.37)	−0.02 (−0.74–0.69)
60	1.38 ± 0.14	1.25 ± 0.14	1.28 ± 0.13	0.72 (−0.02–1.46)	−0.26 (−0.98–0.45)
80	1.18 ± 0.14	1.06 ± 0.13	1.11 ± 0.14	0.46 (−0.27–1.18)	−0.38 (−1.10–0.34)
100	1.14 ± 0.14	1.02 ± 0.11	1.06 ± 0.13	0.57 (−0.16–1.30)	−0.32 (−1.04–0.40)
**Load (%)**	**PBV**				
	**TENDO**	**PUSH**	**GA**	**TENDO-GA *g***	**PUSH-GA *g***
20	3.09 ± 0.29	2.86 ± 0.26	2.98 ± 0.30	0.36 (−0.37–1.08)	−0.42 (−1.14–0.30)
40	2.71 ± 0.24	2.56 ± 0.25	2.60 ± 0.25	0.46 (−0.27–1.18)	−0.14 (−0.85–0.58)
60	2.32 ± 0.16	2.17 ± 0.16	2.25 ± 0.17	0.45 (−0.28–1.17)	−0.43 (−1.15–0.30)
80	1.95 ± 0.20	1.81 ± 0.19	1.92 ± 0.20	0.16 (−0.56–0.87)	−0.52 (−1.25–0.21)
100	1.84 ± 0.16	1.71 ± 0.12	1.80 ± 0.15	0.28 (−0.44–1.00)	−0.61 (−1.34–0.12)

Notes: Load = % 1RM hang power clean; MBV = mean barbell velocity; PBV = peak barbell velocity.

**Table 5:** Effect size magnitudes (*g*) and 95% confidence intervals for the Tendo Power Analyzer (TENDO) and Push Band 2.0 (PUSH) during the hang high pull compared to the GymAware Powertool (GA).

**Load (%)**	**MBV**				
	**TENDO**	**PUSH**	**GA**	**TENDO-GA *g***	**PUSH-GA *g***
20	1.92 ± 0.27	1.77 ± 0.36	1.80 ± 0.25	0.45 (−0.28–1.17)	−0.08 (−0.80–0.63)
40	1.69 ± 0.19	1.49 ± 0.23	1.60 ± 0.18	0.43 (−0.29–1.16)	−0.55 (−1.28–0.18)
60	1.44 ± 0.16	1.26 ± 0.17	1.38 ± 0.16	0.31 (−0.41–1.03)	−0.75 (−1.49–−0.01) *
80	1.21 ± 0.13	1.06 ± 0.14	1.17 ± 0.13	0.32 (−0.40–1.04)	−0.76 (−1.50–−0.02) *
100	1.11 ± 0.14	0.95 ± 0.12	1.06 ± 0.12	0.32 (−0.40–1.04)	−0.86 (−1.61–−0.11) *
**Load (%)**	**PBV**				
	**TENDO**	**PUSH**	**GA**	**TENDO-GA *g***	**PUSH-GA *g***
20	3.08 ± 0.30	2.80 ± 0.34	2.96 ± 0.36	0.35 (−0.37–1.07)	−0.43 (−1.16–0.29)
40	2.67 ± 0.17	2.45 ± 0.21	2.55 ± 0.20	0.64 (−0.10–1.37)	−0.47 (−1.19–0.26)
60	2.33 ± 0.17	2.12 ± 0.18	2.26 ± 0.18	0.38 (−0.34–1.10)	−0.76 (−1.50–−0.02) *
80	2.02 ± 0.18	1.82 ± 0.21	1.99 ± 0.20	0.15 (−0.57–0.87)	−0.82 (−1.56–−0.07) *
100	1.88 ± 0.15	1.67 ± 0.16	1.85 ± 0.16	0.24 (−0.48–0.96)	−1.07 (−1.84–−0.31) *

Notes: Load = % 1RM hang power clean; MBV = mean barbell velocity; PBV = peak barbell velocity; * = indicates a practically meaningful difference compared to the GymAware Powertool.

## Data Availability

The data presented within the current study may be available upon request.
